# Supplementary material for: First Line Systemic Treatment for MALT Lymphoma—Do We Still Need Chemotherapy? Real World Data from the Medical University Vienna
Source: Cancers (Basel). 2020 Nov 26;12(12):3533. doi: 10.3390/cancers12123533 (PMC7761357; doi:10.3390/cancers12123533)
Supplement: Supplementary file 1 [file cancers-12-03533-s001.pdf]

Supplementary Materials: First Line Systemic Treatment for MALT Lymphoma—Do We Still Need Chemotherapy? Real World Data From the Medical University Vienna

Barbara Kiesewetter, Ingrid Simonitsch-Klupp, Marius E Mayerhoefer, Werner Dolak, Julius Lukas and Markus Raderer

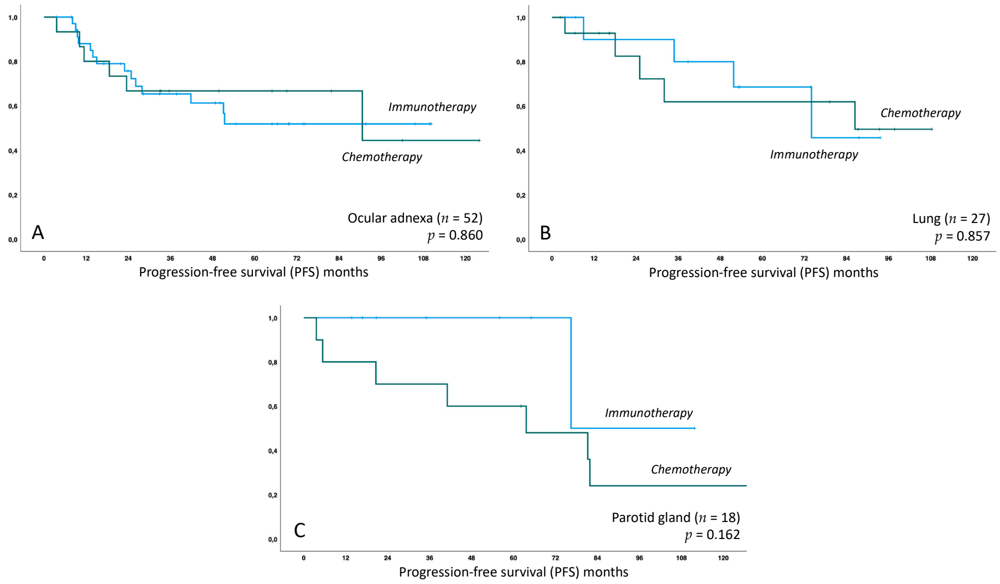

Figure S1. Kaplan-Meier curve for estimated progression-free survival in MALT lymphoma patients treated with immunotherapy versus chemotherapy for further common primary localizations: (**A**) ocular adnexal MALT lymphoma patients; (**B**) pulmonary MALT lymphoma patients; (**C**) parotid gland MALT lymphoma patients. *X*-axis follow-up in months, *Y*-axis cumulative progression-free survival.

Table S1. Univariate analysis for PFS and OS according to clinical features.

Clinical Features	P-Value PFS	P-Value OS
Sex (female /male)	$p = 0.568$	$p = 0.936$
Age 70+	$p = 0.613$	*p = 0.022*
MALT-IPI > 0	$p = 0.614$	$p = 0.119$
Gastric versus extragastric	$p = 0.196$	$p = 0.654$
Localized versus disseminated	$p = 0.163$	$p = 0.129$
Autoimmune disorder	$p = 0.078$	$p = 0.740$
LDH > ULN	*p = 0.010*	$p = 0.331$
Performance status > 1	$p = 0.492$	*p = 0.024*
Plasmacytic diff.	$p = 0.912$	$p = 0.390$
Beta2-microglobulin > ULN	$p = 0.981$	*p = 0.010*
Paraproteinemia	$p = 0.379$	*p = 0.009*
Viral hepatitis	$p = 0.211$	$p = 0.500$

Italics indicate significance. *Abbreviations in chronological order:* PFS = progression-free survival; OS = overall survival; MALT = mucosa associated lymphoid tissue lymphoma; IPI = international prognostic index; LDH = lactate dehydrogenase; ULN = upper limit of normal; diff. = differentiation.

Publisher's Note: MDPI stays neutral with regard to jurisdictional claims in published maps and institutional affiliations.

© 2020 by the author. Licensee MDPI, Basel, Switzerland. This article is an open access article distributed under the terms and conditions of the Creative Commons Attribution (CC BY) license (http://creativecommons.org/licenses/by/4.0/).
